# Evaluating the Effects of Coping Style on Allostatic Load, by Sex: The Jackson Heart Study, 2000–2004

**DOI:** 10.5888/pcd12.150166

**Published:** 2015-10-01

**Authors:** Cristina A. Fernandez, Eric B. Loucks, Kristopher L. Arheart, DeMarc A. Hickson, Robert Kohn, Stephen L. Buka, Annie Gjelsvik

**Affiliations:** Author Affiliations: Eric B. Loucks, Department of Epidemiology, Brown University School of Public Health, Providence, Rhode Island; Kristopher L. Arheart, Department of Public Health Sciences, University of Miami Miller School of Medicine, Miami, Florida; DeMarc A. Hickson, Department of Medicine, University of Mississippi Medical Center and Center for Research, Evaluation, and Environmental and Policy Change, My Brother's Keeper, Inc, Jackson, Mississippi; Robert Kohn, Warren Alpert Medical School of Brown University, Department of Psychiatry and Human Behavior , Providence, Rhode Island; Stephen L. Buka, Department of Epidemiology, Brown University School of Public Health, Providence, Rhode Island; Annie Gjelsvik, Department of Epidemiology, Brown University School of Public Health and Department of Pediatrics and Hasbro Children’s Hospital, Providence, Rhode Island

## Abstract

The objective of this study was to examine the cross-sectional association between coping styles and allostatic load among African American adults in the Jackson Heart Study (2000–2004). Coping styles were assessed using the Coping Strategies Inventory-Short Form; allostatic load was measured by using 9 biomarkers standardized into *z*-scores. Sex-stratified multivariable linear regressions indicated that females who used disengagement coping styles had significantly higher allostatic load scores (β = 0.016; 95% CI, 0.001–0.032); no such associations were found in males. Future longitudinal investigations should examine why disengagement coping style is linked to increased allostatic load to better inform effective interventions and reduce health disparities among African American women.

## Objective

Allostatic load is a measure of physiological wear and tear on the body’s regulatory systems resulting from a cumulative exposure to stressors (1). Fortunately, buffering stress-mediating factors, such as positive coping styles, may be inversely associated with allostatic load; however, there is little information on the link between coping and allostatic load among African Americans (2,3). Using data from the Jackson Heart Study, we hypothesized that positive coping styles (ie, engagement) are inversely associated with allostatic load, and negative coping styles (ie, disengagement) are positively associated with allostatic load among African American adults, and that the results would vary by sex (3–6).

## Methods

Data were obtained by examining the baseline data of the Jackson Heart Study, a cohort of African American adults from the Jackson, Mississippi, tri-county area (N = 5,301; women, n = 3,360; men, n = 1,941). The Jackson Heart Study has been described in detail elsewhere (5). We excluded 2,059 study participants (39%) because of missing information on coping style or allostatic load.

Biomarkers used to conceptualize allostatic load were neuroendocrine (cortisol, ug/dL); metabolic (glycosylated hemoglobin A1c [%]; total cholesterol–high density lipoprotein cholesterol ratio [mg/dL]; waist circumference [cm]); autonomic (systolic blood pressure [SBP] [mmHg]; diastolic blood pressure [DBP] [mmHg]; heart rate [beats/min]); and immune (C-reactive protein [mg/dL]; white blood cell count [th/cmm]) (4,5). We standardized each biomarker into *z* scores, created domain-specific measures, and averaged the *z* scores to create a global allostatic load measure. Higher allostatic load scores indicate poorer overall functioning (1).

Coping styles were measured by using the Coping Strategies Inventory Short Form, a validated 16-item instrument used to measure engagement and disengagement coping styles. Engagement occurs when a person actively confronts a stressor (eg, “I tackle the problem head on.”). Disengagement occurs when a person avoids a stressor (eg, “I try not to think about the problem”). Each item was evaluated by using a 5-point Likert scale (1 = never, 2 = seldom, 3 = sometimes, 4 = often, and 5 = almost always). Scores within each 8-item sub-scale were summed (range: 8–40). Cronbach’s α is 0.59 for the disengagement scale and 0.70 for the engagement scale in the Jackson Heart Study cohort (6).

Covariates were chosen on the basis of previous research (1,3,4): age, education, smoking, alcohol consumption, and physical activity.

All analyses were stratified by sex because of physiological differences of allostatic load by sex (4,5,7,8). Multivariable linear regression analyses were used to determine whether coping styles were associated with allostatic load and its individual components. Analyses were conducted by using STATA/MP version 12.1 (StataCorp LP).

## Results

The analytic sample consisted of 2,068 women (mean age: 53.6 years; SD=12.5 years) and 1,174 men (mean age, 52.7 y; SD, 12.5 y) from the baseline assessment of the Jackson Heart Study (N = 3,242). Men had higher allostatic load scores than women (0.15 vs −0.19; *P* < .001). Coping style scores were similar for both sexes (Table 1).

**Table 1:** Selected Cohort (N = 3,242) Characteristics, Stratified By Sex, Jackson Heart Study, 2000–2004

Characteristic	Men	Women
**Total n, (%)**	1,174 (36.2)	2,068 (63.8)
**Age, y, mean (SD)**	52.7 (12.5)	53.6 (12.5)
**Education, n (%)**		
Less than a high school diploma	180 (15.3)	277 (13.4)
High school diploma or GED	487 (41.5)	879 (42.5)
More than a high school diploma	502 (42.8)	910 (44.0)
Data missing	5 (0.4)	2 (0.1)
**Ever smoked, n (%)**		
Yes	471 (40.1)	508 (24.6)
No	702 (59.8)	1,557 (75.3)
Data missing	1 (0.1)	3 (0.2)
**Consumed alcohol in past 12 months, n (%)**		
Yes	708 (60.3)	815 (39.4)
No	462 (39.4)	1,243 (60.1)
Data missing	4 (0.3)	10 (0.5)
**Physical activity^a^, n (%)**		
Yes	291 (24.8)	393 (19.0)
No	882 (75.1)	1,675 (81.0)
Data missing	1 (0.1)	0 (0)
Allostatic load score^b^, mean (SD)	0.15 (1.43)	−0.19 (1.42)
**Coping styles, mean (SD)**		
Engagement	27.9 (4.5)	28.3 (4.6)
Disengagement	19.2 (4.0)	20.4 (3.9)

Abbreviations: GED, general education development; SD, standard deviation.

a Physical activity was measured as a summary score of the frequency and duration of watching television; walking or bicycling to work, to school, or on errands; and physical exercise (modified from the Baecke physical activity questionnaire) (9).

b Allostatic load levels are presented as *z* scores.

The sex-stratified multivariable linear regression indicated that women reporting higher disengagement coping styles had significantly higher allostatic load scores (β = 0.016; 95% CI, 0.001–0.032; F=14.57), after adjusting for age, education, smoking status, alcohol use, and physical activity. No significant associations were observed among men or among engagement coping styles (Table 2).

**Table 2:** Multivariable Linear Regressions Estimating Allostatic Load (AL) Score by Cohort (N = 3,242) Coping Styles, Stratified by Sex, Jackson Heart Study, 2000–2004

Model	Men		Women	
	Engagement	Disengagement	Engagement	Disengagement
**Model 1: global AL *z* score^a^, β (95% CI)**	0.007 (−0.011 to 0.025)	−0.001 (−0.021 to 0.019)	−0.002 (−0.015 to 0.010)	0.016 (0.001 to 0.032^b^)
Adjusted *R* ^2^	0.045	0.043	0.043	0.044
*F *	8.8^c^	8.66^c^	14.50^c^	14.57^c^
**Model 2, Individual AL components^d^, β (95% CI)**				
Model 2a, Systolic blood pressure	0.004 (−0.008 to 0.016)	0.003 (−0.010 to 0.016)	−0.004 (−0.013 to 0.004)	0.011 (0.001 to 0.020^b^)
Model 2b, Diastolic blood pressure	0.005 (−0.008 to 0.018)	−0.003 (−0.017 to 0.012)	−0.001 (−0.010 to 0.007)	0.010 (0.000 to 0.021)
Model 2c, Heart rate	−0.010 (−0.023 to 0.004)	−0.007 (−0.022 to 0.008)	−0.001 (−0.010 to 0.008)	0.006 (−0.005 to 0.017)
Model 2d, Cortisol	0.006 (−0.006 to 0.018)	−0.003 (−0.017 to 0.010)	0.001 (−0.008 to 0.009)	0.003 (−0.008 to 0.013)
Model 2e, Hemoglobin A1c, %	−0.003 (−0.016 to 0.010)	0.005 (−0.010 to 0.019)	0.009 (0 to 0.018^b^)	−0.002 (−0.012 to 0.009)
Model 2f, Waist circumference	0.005 (−0.007 to 0.017)	0.007 (−0.007 to 0.020)	−0.007 (−0.016 to 0.003)	0.014 (0.003 to 0.025^b^)
Model 2g, Ratio, total cholesterol to HDL cholesterol	0.002 (−0.009 to 0.014)	0.002 (−0.011 to 0.014)	−0.003 (−0.012 to 0.005)	−0.003 (−0.013 to 0.007)
Model 2h, White blood count	−0.004 (−0.021 to 0.013)	−0.005 (−0.024 to 0.013)	0.003 (−0.005 to 0.011)	0.006 (−0.003 to 0.016)
Model 2i, C-reactive protein	0.004 (−0.003 to 0.012)	0.002 (−0.006 to 0.010)	−0.001 (−0.009 to 0.007)	0.003 (−0.006 to 0.013)

Abbreviations: CI, confidence interval; HDL, high density lipoprotein.

a Outcome for model 1 is global allostatic load adjusted for age, education, smoking status, alcohol consumption, and physical activity.

b *P* < .05.

c *P* < .001

d Outcomes for models 2a through 2i are individual allostatic load components adjusted for age, education, smoking status, alcohol consumption, and physical activity.

We further examined the individual allostatic load components within the global allostatic load scores to determine which of the components were the primary drivers of the significant association between disengagement coping styles and allostatic load among women. Results indicated that women who used disengagement styles had significantly elevated SBP (β = 0.011; 95% CI, 0.001–0.02) and waist circumference (β = 0.014; 95% CI, 0.003–0.025). Additionally, women who used engagement styles had significantly elevated glycosylated hemoglobin A1c% (β = 0.009; 95% CI, 0–0.018). All other individual allostatic load components in either sex were not significant (Table 2). This indicated that of the allostatic load components, systolic blood pressure and waist circumference may be the primary drivers of associations; both have effect sizes outside the 95% confidence intervals.

## Discussion

Results indicated that women who avoided stressors (ie, had disengagement coping styles) were likely to have higher allostatic load levels than women who did not. No significant associations were found for men. Because African American women experience double jeopardy (ie, racism and sexism) and other chronic stressors (ie, familial responsibilities), this combination may subsume their ability to cope effectively with daily hassles, thereby increasing their susceptibility to elevated allostatic load (3,10). Examining more comprehensive biomarkers in studies with longitudinal designs, including potential mediators and moderators, may indicate the reasons for sex differences (5,11).

This study has some limitations that should be noted. The cross-sectional design limits our ability to draw causal inferences. Additionally, we had a large proportion of participants with missing information on coping style and allostatic load. Sensitivity analyses comparing those who were and were not included indicated no significant differences in terms of sex or coping styles, but those who were excluded had significantly higher allostatic load scores (0.18 vs −0.07; *P* < .001). However, the results did not markedly differ after we used multiple imputation methods for missing coping data.

This was the first community-based study to examine coping styles and allostatic load in African Americans. African American women who used disengagement coping styles had significantly higher allostatic load scores, which can result in long-term bodily strain (4). Future investigations could consider other potential covariates through which disengagement coping styles increase allostatic load, specifically, SBP and waist circumference, to help inform interventions and reduce health disparities. Additionally, if findings from this study are replicated and extended, it may suggest that teaching positive coping styles to African American women could prevent this maladaptive physiological functioning resulting from increased allostatic load. Because people with positive coping styles are likely to adapt to stressors effectively, using positive coping styles has the potential to build resiliency in African Americans.
